# Recent advances in understanding the mechanisms determining longevity

**DOI:** 10.12688/f1000research.19610.1

**Published:** 2019-08-09

**Authors:** Robert Bayersdorf, Björn Schumacher

**Affiliations:** 1 Institute for Genome Stability in Aging and Disease, Medical Faculty, University of Cologne, Cologne, Germany; 2 Cologne Excellence Cluster for Cellular Stress Responses in Ageing-Associated Diseases (CECAD), Center for Molecular Medicine Cologne, University of Cologne, Cologne, Germany

**Keywords:** Ageing, longevity, DNA repair, autophagy, senescence, C. elegans

## Abstract

The field of aging research has progressed significantly over the past decades. Exogenously and endogenously inflicted molecular damage ranging from genotoxic to organellar damage drives the aging process. Repair mechanisms and compensatory responses counteract the detrimental consequences of the various damage types. Here, we discuss recent progress in understanding cellular mechanisms and interconnections between signaling pathways that control longevity. We summarize cell-autonomous and non-cell-autonomous mechanisms that impact the cellular and organismal aging process

Throughout history, humankind has been preoccupied with longevity, death, and immortality, as evidenced by the first known epic, describing Gilgamesh’s futile quest for immortality. Death due to old age, however, appears to be rather rare in nature, as most species are confronted with various extrinsic sources of mortality, including predation, malnutrition, and life-threatening temperatures, all of which can limit the life span of individuals in their natural habitats. The vastly different life spans among closely related species ^1, 2^ were selected mainly via pressure exerted by extrinsic mortality risks that had to be balanced with the need for successful offspring generation. Some trees may persist thousands of years, whereas some insect species live for only a few days and other species, such as the small freshwater animal *hydra*, are thought to live indefinitely ^3^. Various primate species show considerable life span variations, ranging from around 10 to 60 years, even in protected environments. It is vigorously debated whether humans have a fixed maximum life span that plateaus at 115 years ^4^ or whether the mortality risk plateaus past 105 years ^5^, leaving open the theoretical possibility of immortality—provided there were an infinite number of 105-year-old individuals. In general, not all individuals of a given species reach the same age, even if they live in the same environment. This variation was most impressively demonstrated in a clonal *Caenorhabditis elegans* population that even under identical environmental conditions showed a stochastic life span distribution ^6^. Genome comparisons from species groups with different life spans have revealed evolutionary signatures in genes, some of which have been implicated in pathways associated with longevity regulation ^7, 8^. Among these are genes involved in DNA repair, splicesosome and RNA processing, cell cycle control and cell division, kynurenine metabolism, autophagy, wound healing, and hemostasis ^7, 8^. Such studies offer great potential for identifying aging modulators to enhance our understanding of the factors that govern the dynamics of life span determination. ****

Over the past three decades, environmental and metabolic factors as well as evolutionarily conserved pathways that influence life span have been identified ( Figure 1). Examples include several stress factors that, in excess, can negatively affect life span but that, in moderation, can trigger protective responses that lead to life span extension in a process called hormesis ^9^. For example, DNA damage is thought to accumulate in tissues during aging, as extrinsic and intrinsic sources of genotoxic stress lead to a wide array of DNA lesions, including oxidized DNA bases, apurinic sites, and DNA double-strand breaks (reviewed in 10). DNA damage drives the aging process via mechanisms ranging from interference with replication and transcription to the DNA damage response (DDR) that triggers apoptosis and cellular senescence ^11^. A range of congenital DNA repair defects lead to progeroid syndromes that are characterized by accelerated segmental aging phenotypes in humans ^12, 13^. Reactive oxygen species (ROS) have been prime suspects for accelerating the aging process; however, they can also trigger protective responses at low levels and are even necessary for certain life span extension phenotypes ^14^. For example, epigallocatechin-3-gallate (EGCG), a compound found in green tea ( *Camellia sinensis* L.), leads to ROS production and an extended median life span in *C. elegans*, but this effect is abrogated when the nematodes are treated with the ROS-neutralizing reducing agent *N*-acetylcysteine (NAC) ^15^. Similar positive life span and hormetic effects could be observed with low concentrations of other ROS inducers, such as naphthoquinones and arsenite, in a ROS-dependent manner ^16, 17^. Moreover, the influence of glycogen and glucose on the intracellular glutathione redox system, concomitant ROS scavenging, and life span reduction in long-lived *daf-*2 mutant *C. elegans* highlight the importance of ROS signaling and redox systems for life span control ^18, 19^. Strongly elevated ROS levels, however, induced by higher concentrations of paraquat shorten life span, presumably because of oxidative damage ^15, 17, 19^. A similar relationship can be observed regarding the nutritional state of animals, as severe nutrient and energy limitation can lead to death; however, calorie restriction (CR), dietary restriction (DR), or intermittent fasting has positive effects on life span in several model organisms, and modulation of metabolic parameters in a 2-year human trial showed potential benefits ^20– 22^. These life span extensions can also be triggered via modulation of molecular signaling pathways—for example, insulin-like growth factor (IGF) signaling—or via the inhibition of neuronal circuits involved in nutrient sensing ^23^. Based on information regarding the signaling mechanisms that mediate the life span–extending consequences of CR, pharmacological inhibitors such as the mammalian target of rapamycin (mTOR) inhibitor rapamycin were shown to be sufficient for life span extension in several model organisms, including mice ^24^. Currently, several efforts are underway to develop more specific TORC1 inhibitors to avoid the side effects associated with rapamycin treatment in humans, such as immunosuppression and impaired wound healing ^25– 27^.

**Figure 1.**
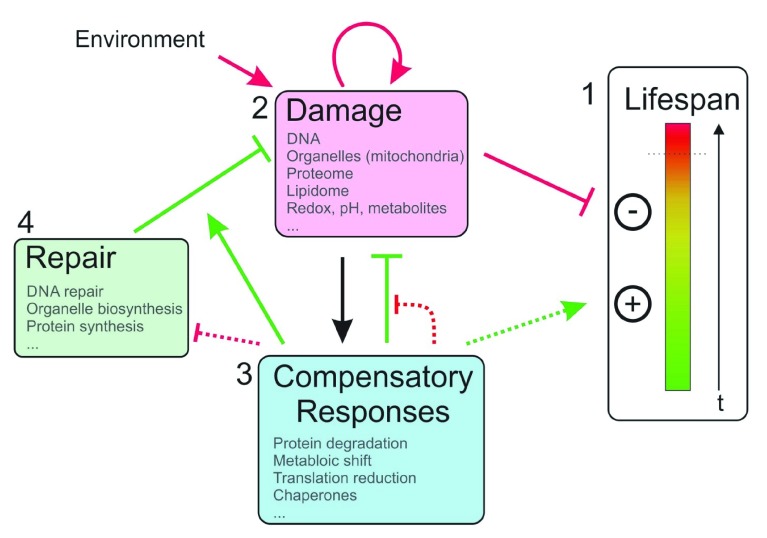
Life span determination. (1) Organismal life starts out as a system in healthy homeostasis (green bar area), which becomes increasingly disorganized via deleterious effects (yellow/red bar area) until it reaches a threshold of system collapse and death (dotted line). Positive and negative effects determine the dynamics of this transition and therefore the life span of the individual. (2) Biological damage leading to disruption of systemic homeostasis can be triggered by environmental insults and internal metabolic processes, which can self-amplify and interact which each other. (3) Damage triggers compensatory responses that limit damage (green blunt arrow), facilitate damage repair (solid green arrow), and delay the complete disruption of homeostasis (dotted green arrow). Over time, compensatory responses exhaust their compensatory capacity and potentially limit repair resources (dotted red arrows). (4) Repair and re-synthesis of biological structures and components at least partially revert some types of damage. Green arrows denote positive effects on life span, red arrows denote negative effects on life span, and black arrows denote neutral or ambiguous effects.

Protein homeostasis, also called proteostasis, describes a compendium of processes that include proteasome-dependent protein degradation, several types of autophagy required for the degradation of biomolecules, protein aggregates and defective organelles (reviewed in 28), as well as downregulation of ribosomal protein translation ^29, 30^. Enhanced proteostasis mechanisms are often essential components of lifespan-extending pathways ( Figure 1). Inhibition of TOR signaling, for instance, reduces the initiation of protein translation, which might then alleviate proteotoxic stress, e.g., the age-related accumulation of protein aggregates ^30^. In addition, shifts in overall metabolism might cause or result from changes in proteostatic capacity ^31^. For example, mitochondria, which have important roles in energy generation and act as hubs for lipid metabolism and anabolic processes, have been implicated in lifespan regulation. Treatment with the natural compound urolithin A induces mitophagy and extends lifespan in *C. elegans* perhaps by eliminating dysfunctional mitochondria, thus linking autophagy and mitochondrial physiology to life span determination ^32^. Their involvement might be context-dependent, as mitochondrial stress responses, such as the mitochondrial unfolded protein response (UPR ^mt^), can contribute to life span extension in *C. elegans* ^23^. In contrast, life span–extending mutations in the electron transport chain complex IV (COX-5B, previously referred to as CCO-1) or cytochrome *c* reductase (ISP-1) induce UPR ^mt^ marker expression; however, abrogating this induction via mutation of the transcription factor (TF) ATFS-1 does not ameliorate this life span extension ^33^. Zhou *et al*. demonstrated that the consequence of autophagy can also be context-dependent: whereas increased autophagy is necessary for many life span–extending paradigms (that is, CR), increased mitochondrial permeability can convert elevated autophagy into a life span–shortening process in *C. elegans* ^34^. This observation suggests that mitochondrial and autophagic functions may interact to modulate longevity.

A range of TFs and epigenetic and splicing modulators ^35^ that regulate stress responses and confer positive effects on life span have been identified. The discovery that attenuation of the insulin-like signaling (IIS) pathway extends life span in *C. elegans* ignited the field of the genetics of longevity. Importantly, IIS regulates life span via activation of the FOXO TF DAF-16. Since these seminal discoveries, numerous additional life span–regulating TFs have been identified. The HLH-30/TFEB TF mediates longevity via stress-induced regulation of autophagy-associated genes upon stress. Although these TFs have been characterized in isolation for quite some time, how they can cooperate with a variety of other TFs to regulate life span and stress responses has been recently delineated. Lin *et al*. suggested that DAF-16/FOXO and HLH-30/TFEB cooperate to regulate longevity and also have important independent functions under specific stress conditions ^36^. In contrast, the homeodomain TF CEH-60/PBX, whose role in development has been investigated, was recently shown to negatively affect life span in *C. elegans* by repressing DAF-16 activity ^37^. HLH-30 activity, which has positive effects on life span, can be enhanced via treatment with nuclear export blocker drugs ^38^ and modulators of intracellular calcium storage compartments ^39^. On the level of mRNA processing, the splicing factor SFA-1/SF1 has emerged as a requirement for the proper splicing of transcripts involved in lipid metabolism and other metabolic processes under conditions of life span–extending DR ^35^.

Epigenetic mechanisms are crucial for cell identity and function of differentiated cells, the differentiation of stem cells, and stem cell maintenance. So far, different adult stem cell systems have been identified which show aging-associated changes in epigenetic marks on chromatin and DNA (for example, in hematopoietic stem cells [HSCs], intestinal stem cells, and muscle stem cells [MuSCs]). Cell-intrinsic damage, accumulating during stem cell quiescence, may lead to epigenetic changes, while cell elimination is prevented through anti-apoptotic pathways (reviewed in 40). For example, these changes can lead to divergent deposition of H3K4me3 marks or action of Rad21/cohesin, resulting in impaired stem cell function and senescence signaling in aged MuSCs ^41^ or over-activation of inflammatory signaling in HSCs ^42^, respectively. Deficiencies in stem cell function, in turn, impair tissue homeostasis, function, and regeneration, leading to declining organismal health and most likely limiting life span. Moreover, epigenetic changes in aged stem cells might lead to increased incidences of aging-related diseases such as hematopoietic cancers by allowing the clonal selection of mutated HSCs ^43– 45^. Interestingly, transient epigenetic reprogramming via cyclic expression of the Yamanaka factors in mice showed signatures of improved health in a progeroid laminopathy mouse model ^46^.

It is becoming increasingly clear that in addition to cell-autonomous stress responses that modulate the intrinsic resilience of individual cells upon exposure to specific types of stress, signaling between cells and tissues can elicit such responses both locally and in distal tissues. The influence of these inter-tissue effects has been exemplified by heterochronic parabiosis and transplantation experiments, which suggest differences in circulating and local niche factors to dynamically alter tissue functionality and epigenetic states during aging ^40, 47, 48^. In particular, neuronal tissue has come into focus as a significant coordinator of life span–modulating processes; for example, neuronal autophagy (as well as autophagy in the intestine) can regulate life span–extending processes non-cell-autonomously ^49^. The immune system is an important non-cell-autonomous regulator that not only profoundly influences life span directly by preventing premature death due to infections but also protects organisms via cancer surveillance and removal of senescent cells. While the prowess of the immune system fades during aging through a process called immunosenescence ^50^, nuclear DNA damage, accumulating extranuclear DNA, and senescent cells fuel inflammation ^51^. Parts of the adaptive immune repertoire of individual aging humans have recently been characterized, and declines in the diversity of CD8 ^+^ (cytotoxic) T-cell and B-cell repertoires were observed ^52, 53^. Senescent cells, which adopt a distinct physiology (often via continuous DNA damage signaling ^11^), have themselves been implicated in impairing normal tissue function and promoting the development of a deleterious, chronic pro-inflammatory environment ^54– 56^. Targeting senescent cells has shown positive effects on immune function in mice and therefore appears to be a promising field of research to improve tissue aging in the elderly ^57^, including attempts to re-establish a balanced output of aging HSCs to regenerate lymphopoiesis during aging ^58^. In contrast, the senescence program might protect cells from transforming into cancer cells and has been implicated in tissue regeneration after skin injury ^59^. Together, these observations indicate that senescent cells serve dual roles in influencing life span: pro-longevity tumor suppression and tissue repair versus involvement in pro-aging inflammatory reactions.

As the important roles for the immune system and metabolism in life span modulation have been intensely studied for some time, the involvement of the microbiome has emerged more recently. Different microbiota compositions have been shown to modulate both the immune system and metabolism in positive and pathogenic ways. Studies on centenarians have suggested specific signatures in the gut microbiome in terms of its composition and diversity in long-lived humans ^60, 61^. For example, higher relative abundances of *Akkermansia* and *Bifidobacterium*, known health-associated microbes, are positively associated with exceptionally long-lived humans ^60^. Furthermore, microbial transfer experiments from young to middle-aged killifish, a short-lived model organism recently established for conducting research on vertebrate aging, demonstrated a positive effect of a young microbiome on life span ^62^.

Many stress factors that influence life span (for example, ROS, mitochondrial impairment, cellular redox imbalance, nutritional status, and protein translation) are intimately connected ( Figure 1). Thus, the delineation of clear cause-and-effect relationships between such factors and longevity is challenging. ROS, for example, can cause proteome stress ^63^ and lead to DNA damage ^64^ among other effects; however, it also stimulates protective (hormetic) responses ^9^. DNA damage, in turn, can lead to more ROS production ^65^, potentially also via consequential imbalances in the mitochondrial proteome. The DDR, which comprises checkpoint signaling and DNA repair pathways, protects cells from malignant transformation ^66^. The DDR can also lead to decreased general translation ^67^, which might alleviate proteostatic stress. The involvement of proteostasis in the DDR appears manifold, ranging from distinct roles during DNA repair to ensuring cellular homeostasis. DNA damage remains a central node in the network of these processes, as both exogenous and endogenous genotoxins constantly inflict DNA damage and because the DDR affects a vast range of metabolic and proteostatic responses ^68^. Interestingly, CR was shown to dramatically extend life span in nucleotide excision repair–deficient progeroid animals ^69^, suggesting a new perspective in the ongoing quest for therapies for congenital progeroid syndromes. Indeed, DAF-16–mediated stress responses to DNA damage in *C. elegans* can preserve tissue maintenance and function by elevating the tolerance to persistent DNA damage ^70^.

Interventional studies with the mTOR inhibitor rapamycin have had positive effects on delaying the onset of age-associated chronic disease markers and potentially negative effects in humans ^71– 73^. In addition to rapamycin, the drugs resveratrol and metformin have been used to modulate pathways involved in DR-mediated life span extension, and senolytic drugs (for example, dasatinib, quercetin ^74^, and fisetin ^75^) have been reported to differentially enhance apoptosis in senescent cells, depending on their original cell type. (Senolytics are reviewed in 76.) Dasatininb and quercetin were recently tested in a first human pilot study on idiopathic pulmonary fibrosis; however, the long-term effects of these treatments have not been assessed ^77^. These interventions might indeed provide potential therapeutic options for delaying the aging process; however, drugs for specifically enhancing DNA repair or alleviating DNA damage have not been developed yet. In contrast, the deletion of DDR components was shown to exert positive effects on tissue maintenance and life span in mice that prematurely aged because of telomere dysfunction ^78– 80^. These results suggest that modulating the DDR could provide interesting avenues for interventions for maintaining tissue homeostasis during aging.

In summary, recent progress has significantly expanded our knowledge of the various processes that modulate life span, including genetic regulators, stress responses, metabolism, cellular senescence, and inter-tissue communication. Stochastic effects, such as the occurrence of DNA damage, which can impact each one of those processes ^68^ could result in different individual aging trajectories, where hypothetical tipping points of declining tissue functionality are reached at different time points ^81, 82^. This might be one reason for the heterogeneity of individual life spans even in defined, homogenous model organism populations. Importantly, the interactions between longevity modulators are becoming increasingly apparent, highlighting the complexities underlying aging and life span determination. As multiple factors contribute to aging and the alleviation of age-related organismal deterioration, it might be necessary for future interventions to collectively target a range of longevity modulators—potentially even in a tissue- or cell type-specific manner—to extend the healthy life span in humans.
